# Supplementary material for: Drosophila maintain a consistent navigational goal angle for days to weeks
Source: bioRxiv. 2025 Dec 11:2025.12.09.693277. Preprint. [Version 1] doi: 10.64898/2025.12.09.693277 (PMC12714015; doi:10.64898/2025.12.09.693277)
Supplement: 1 [file NIHPP2025.12.09.693277v1-supplement-1.pdf]

bioRxiv preprint doi: https://doi.org/10.64898/2025.12.09.693277; this version posted December 11, 2025. The copyright holder for this preprint (which was not certified by peer review) is the author/funder, who has granted bioRxiv a license to display the preprint in perpetuity. It is made available under aCC-BY 4.0 International license.

Supplemental Figures

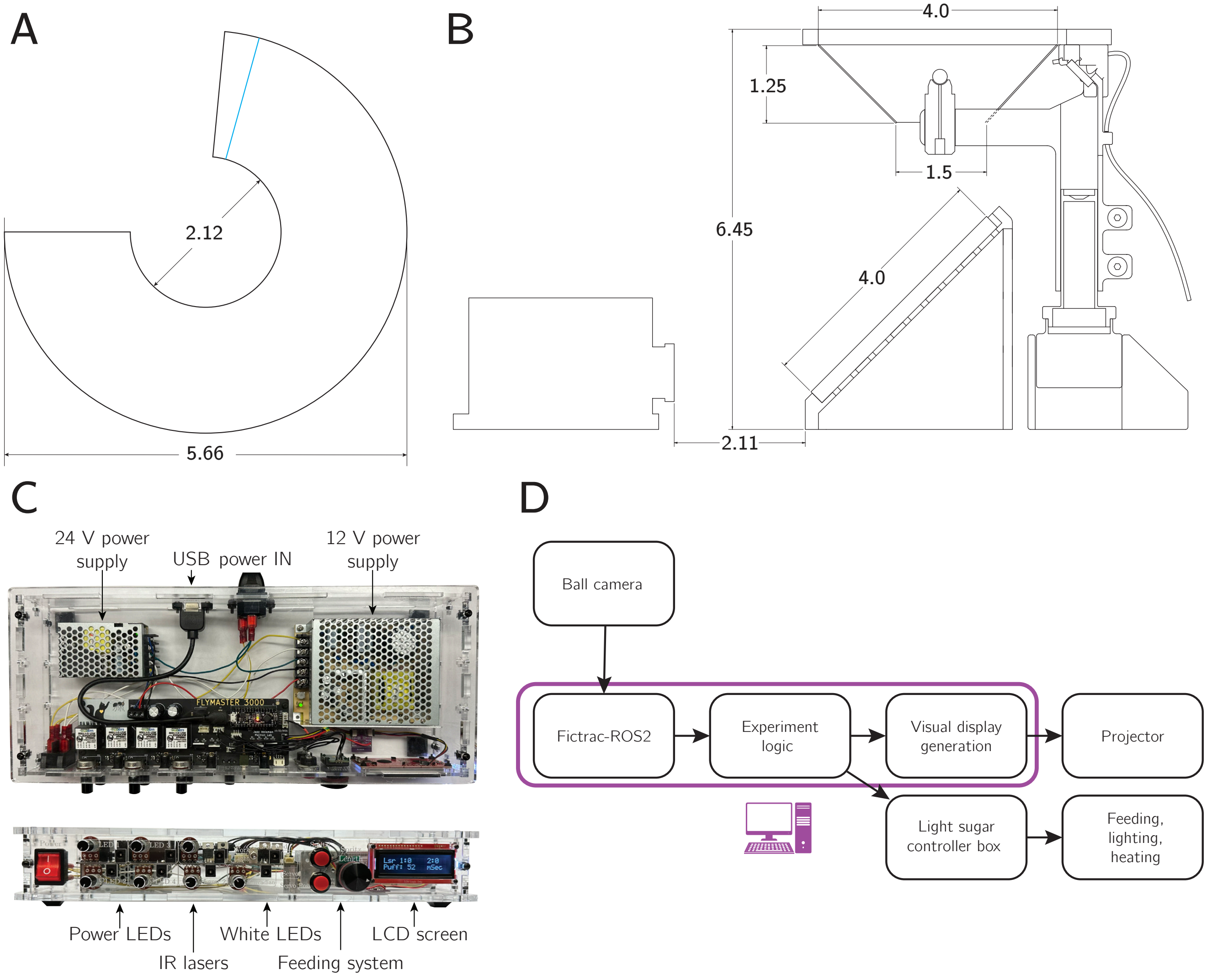

Figure S1: **A**: The template for cutting out the conical screen. The blue line marks where the opposite edge should line up to form the correct frustum shape, and can be engraved with the laser cutter to mark it. All dimensions are in inches. **B**: Dimensional drawing of the display assembly. **C**: Top and front views of a controller box that drives electronic components on the rig. **D**: Schematic of information flow through the rig

bioRxiv preprint doi: https://doi.org/10.64898/2025.12.09.693277; this version posted December 11, 2025. The copyright holder for this preprint (which was not certified by peer review) is the author/funder, who has granted bioRxiv a license to display the preprint in perpetuity. It is made available under aCC-BY 4.0 International license.

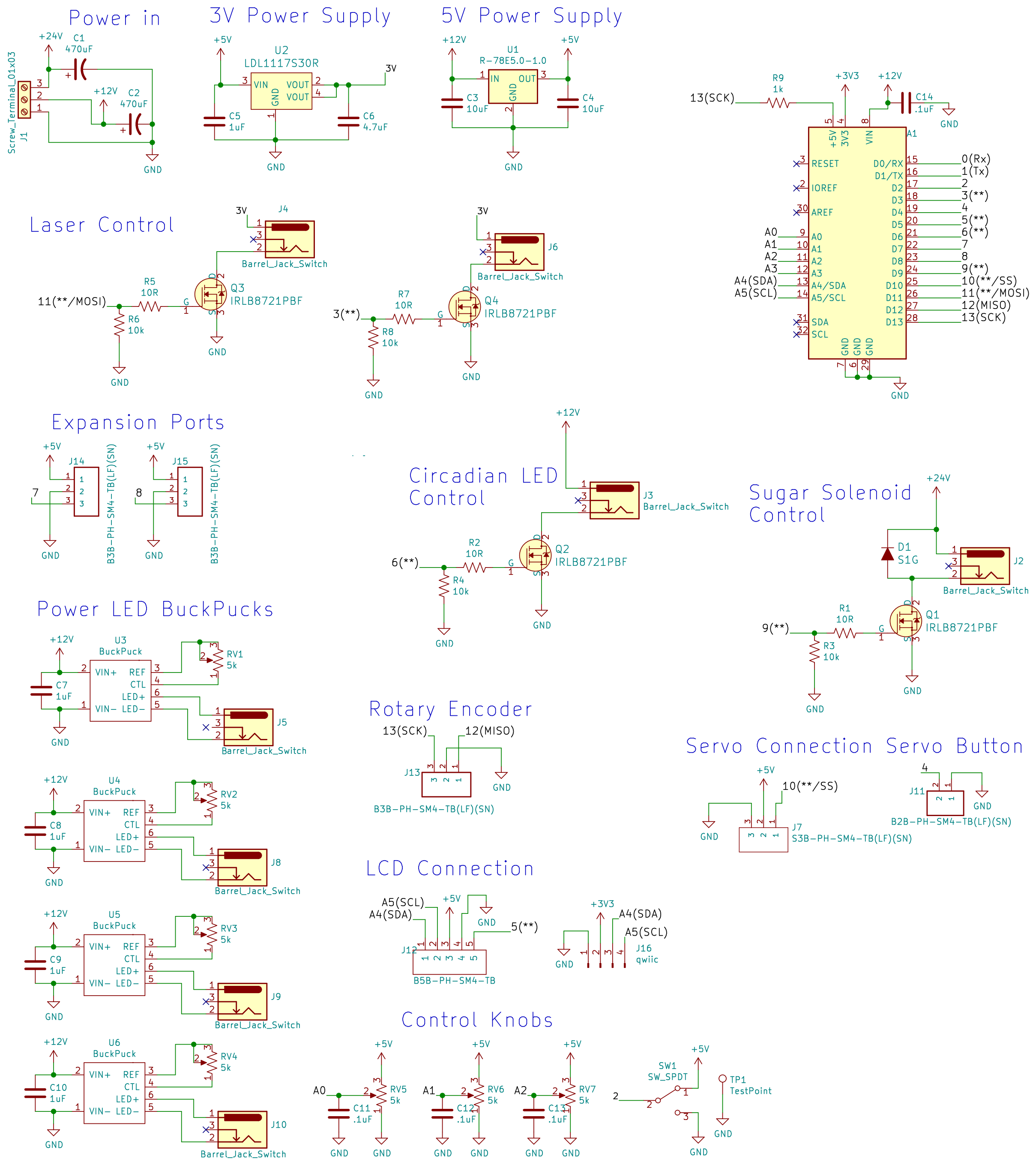

Figure S2: Schematic for the controller box PCB.

bioRxiv preprint doi: https://doi.org/10.64898/2025.12.09.693277; this version posted December 11, 2025. The copyright holder for this preprint (which was not certified by peer review) is the author/funder, who has granted bioRxiv a license to display the preprint in perpetuity. It is made available under aCC-BY 4.0 International license.

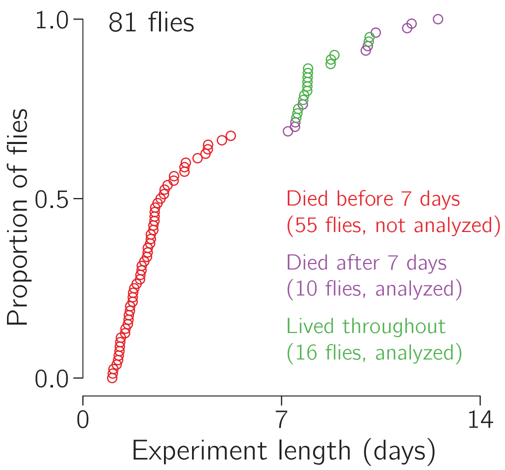

Figure S3: Survival curve for the flies analyzed in Figures 2 and 3 Each fly's survival time is indicated by a circle. Red circles indicate flies that did not live long enough to be included. Purple circles indicate flies were included but died while on the rigs. Green circles indicate flies that were removed from the rigs without dying.

bioRxiv preprint doi: https://doi.org/10.64898/2025.12.09.693277; this version posted December 11, 2025. The copyright holder for this preprint (which was not certified by peer review) is the author/funder, who has granted bioRxiv a license to display the preprint in perpetuity. It is made available under aCC-BY 4.0 International license.

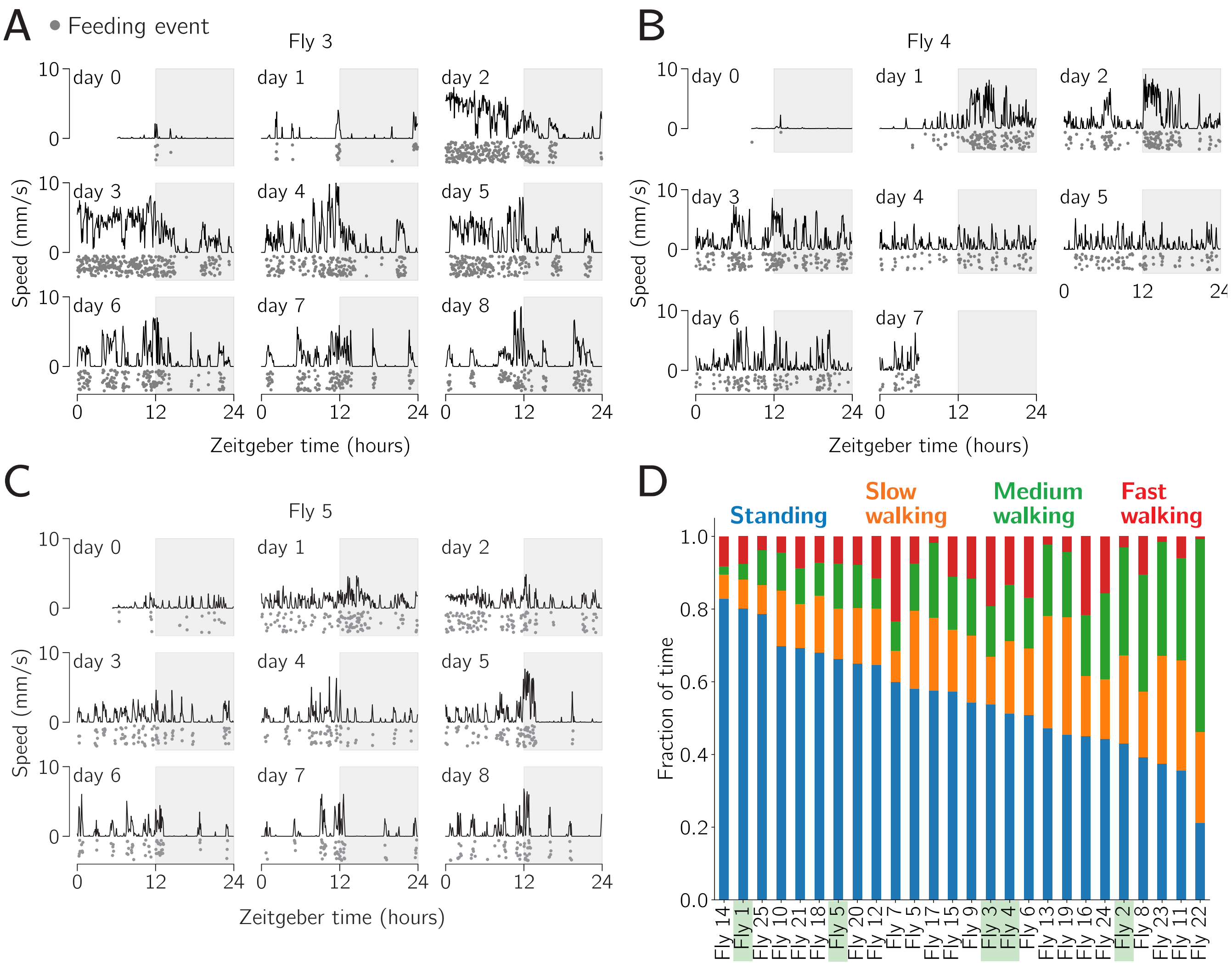

Figure S4: Individual flies show different walking dynamics. **A-C:** Translational speed over time for three example flies (1 min bins). Each feeding event is indicated by a gray dot below the speed trace (each dot's y position is jittered for clarity). **D:** Binned translational speed profiles for all 26 flies in the data set. For each fly, the mean translational speed was calculated in each minute and assigned to a behavioral bin as follows: standing (<0.1 mm/s), slow (0.1 – 1 mm/s), medium (1 – 3 mm/s), or fast (>3 mm/s). Flies are sorted by the standing fraction. The example flies from panels A-C, Figure 2 C-D, and Figure 3 B and D, are highlighted in green.

bioRxiv preprint doi: https://doi.org/10.64898/2025.12.09.693277; this version posted December 11, 2025. The copyright holder for this preprint (which was not certified by peer review) is the author/funder, who has granted bioRxiv a license to display the preprint in perpetuity. It is made available under aCC-BY 4.0 International license.

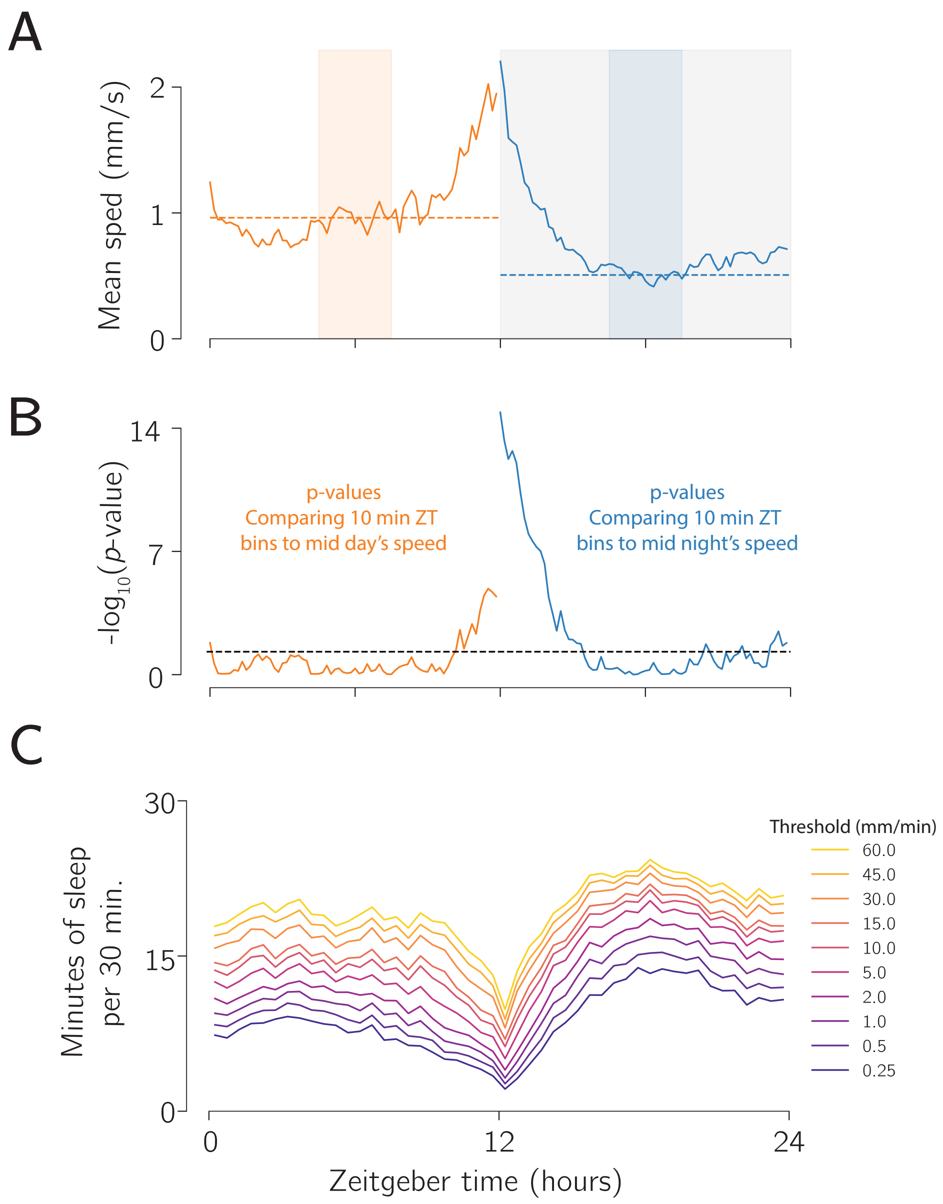

Figure S5: Flies walk significantly faster in anticipation of the lights turning on and off. **A:** Mean speed across all flies as a function of Zeitgeber time. The middle three hours of the day (orange shaded region) and night (blue region) form the comparison group for the rest of the day / night timepoints. The mean speed for each is comparison group is shown as a dotted line. **B:** Plot of the $-\log_{10}$ of the p value for the comparison of the distribution of mean speeds for each 10 minute bin compared with the mid-day or mid-night time window (Mann-Whitney U test). Significance threshold of $p = 0.05$ (not corrected for multiple comparisons) is shown as a dashed line. **C:** Average sleep per 30 minute bin parsed by the speed threshold used for defining a standing event.

bioRxiv preprint doi: https://doi.org/10.64898/2025.12.09.693277; this version posted December 11, 2025. The copyright holder for this preprint (which was not certified by peer review) is the author/funder, who has granted bioRxiv a license to display the preprint in perpetuity. It is made available under aCC-BY 4.0 International license.

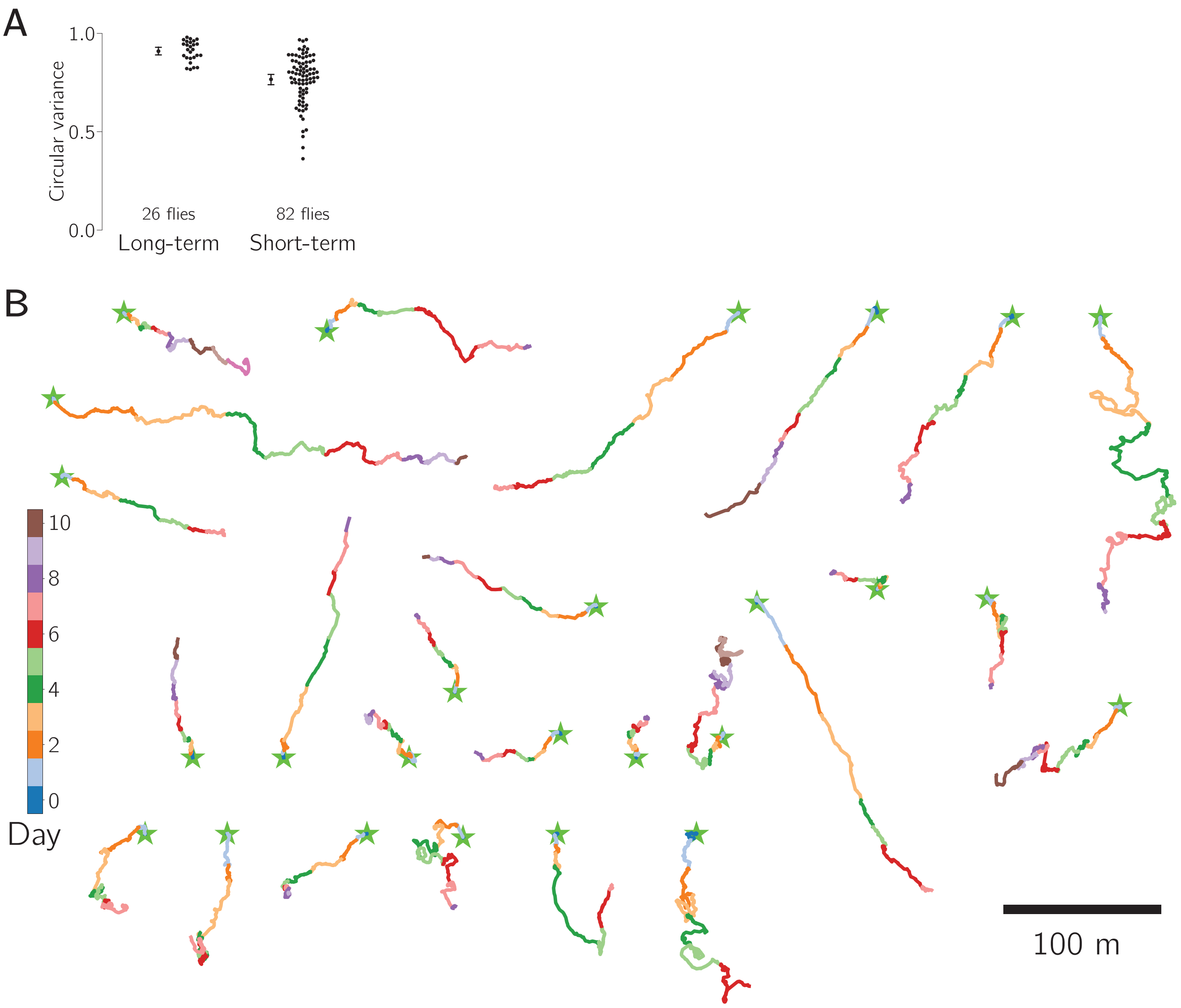

Figure S6: Circular variance analysis and separated trajectories of long-term menotaxis behavior, relating to Figure 3 A: Flies performing long-term menotaxis show significantly higher circular variance in their headings angle while walking compared to flies performing short-term menotaxis. **B:** All trajectories from Figure 3 F, shown separated and colored by day of the experiment. Trajectory start locations are shown as green stars.

bioRxiv preprint doi: https://doi.org/10.64898/2025.12.09.693277; this version posted December 11, 2025. The copyright holder for this preprint (which was not certified by peer review) is the author/funder, who has granted bioRxiv a license to display the preprint in perpetuity. It is made available under aCC-BY 4.0 International license.

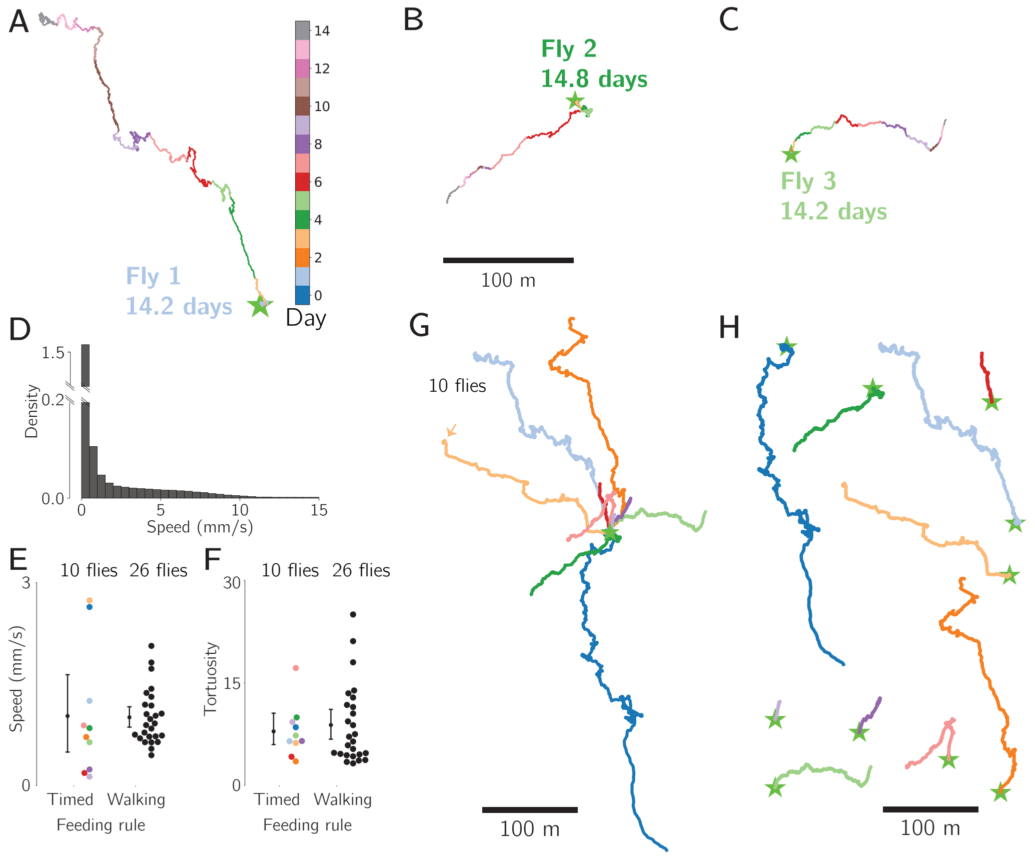

Figure S7: Flies fed every 15 min expressed a preferred traveling direction for up to two weeks. **A, B, C:** 14-day trajectories from three flies. These flies were fed a sugar drop every 15 min. The start of each trajectory is indicated with a green star in all panels. **D:** Histogram of average walking speed calculated in 1 s bins for all flies (standing events excluded). Bin width: 0.5 mm/s. Note discontinuous axis on the first bin. **E:** Comparison of average speed per fly from this data set (timed feeding) with the long-term menotaxis flies from Figure 3 D (walking-triggered feeding). The average speed per fly was not significantly different (Mann-Whitney U test, $p = 0.4$). Mean and bootstrapped 95% confidence intervals are shown. **F:** Tortuosity for each fly's trajectory from this data set (timed feeding) and the flies performing long-term menotaxis flies from Figure 3 D (walking-triggered feeding). The tortuosity per fly was not significantly different between the two groups (Mann-Whitney U test, $p = 0.99$). **G:** Trajectories of all flies from this data set. Individual fly colors match those in panels E, F, and H. All flies were tested for 10–15 days, except for the fly plotted in light orange, and indicated with an arrow, which was tested for just over 5 days. **H:** Same trajectories as in panel G, but shown displaced from one another.

bioRxiv preprint doi: https://doi.org/10.64898/2025.12.09.693277; this version posted December 11, 2025. The copyright holder for this preprint (which was not certified by peer review) is the author/funder, who has granted bioRxiv a license to display the preprint in perpetuity. It is made available under aCC-BY 4.0 International license.

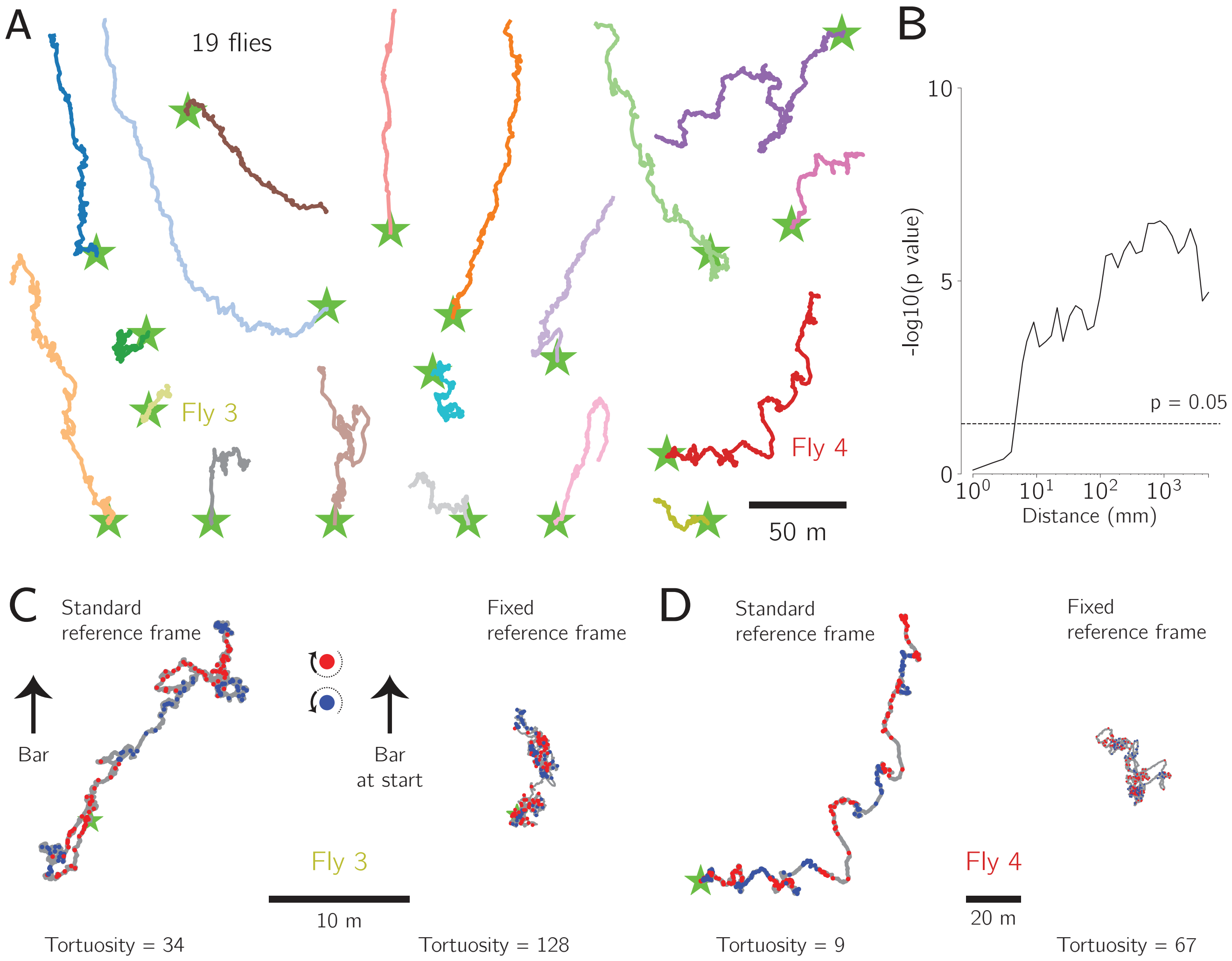

Figure S8: Additional analyses of the virtual rotation experiment. **A:** Entire trajectories for all 19 flies tested in the virtual rotation experiment of Figure 5. Colors associated with individual flies here match those used in Figure 5 E and F. **B:** Plot of the $-\log_{10}(p)$ value vs path distance for the analysis described in Figure 5 C. The dashed line indicates the $p = 0.05$ significance threshold (not corrected for multiple comparisons). **C:** Comparison of the trajectory of an additional example fly in the standard vs fixed reference frame plots of Figure 5. Despite the fact that this example fly had a more tortuous trajectory in the standard frame (tortuosity 34), her trajectory was still even more tortuous in the fixed frame (128). **D:** Same as panel C, for one more fly.

bioRxiv preprint doi: https://doi.org/10.64898/2025.12.09.693277; this version posted December 11, 2025. The copyright holder for this preprint (which was not certified by peer review) is the author/funder, who has granted bioRxiv a license to display the preprint in perpetuity. It is made available under aCC-BY 4.0 International license.

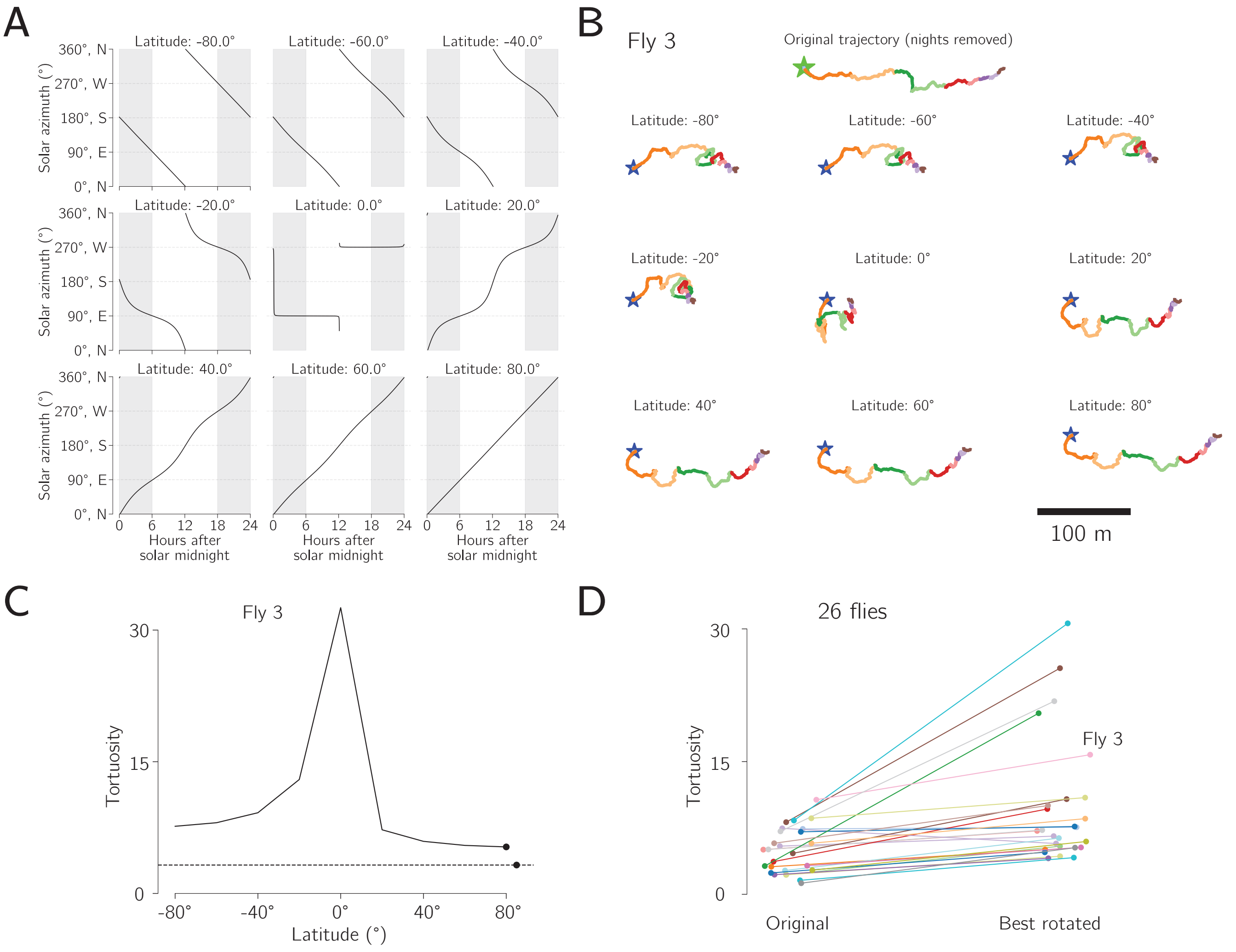

Figure S9: No evidence for circadian time compensation in long-term menotaxis. **A:** Solar ephemeris functions for nine different latitudes, calculated for the equinox. Each plot shows the solar azimuthal angle (degrees) as a function of time after solar midnight. Night time is shaded in gray. **B:** One example fly's trajectory with nights removed (original, top), and the same trajectory rotated according to the solar ephemeris function for each of the nine latitudes (bottom nine plots), labeled by latitude. **C:** Tortuosity of the fly from panel B's trajectory (original, dashed line) compared to the tortuosities of the nine solar-rotated trajectories. **D:** Comparison of the tortuosity of all flies original trajectories vs the lowest tortuosity achieved by rotating according to any of the nine solar ephemeris functions. Tortuosities are significantly higher after solar-rotation manipulations (Wilcoxon signed-rank test, $p = 1.3 \times 10^{-5}$). All but one fly showed an increase in tortuosity after all solar-rotation manipulations.
